# Nanodiamonds for Medical Applications: Interaction with Blood in Vitro and in Vivo

**DOI:** 10.3390/ijms17071111

**Published:** 2016-07-12

**Authors:** Lin-Wei Tsai, Yu-Chung Lin, Elena Perevedentseva, Andrei Lugovtsov, Alexander Priezzhev, Chia-Liang Cheng

**Affiliations:** 1 Department of Physics, National Dong Hwa University, Hualien 97401, Taiwan; siegfriedlenz7974@hotmail.com (L.-W.T.); adam7319@gmail.com (Y.-C.L.); elena@mail.ndhu.edu.tw (E.P.); 2 International Laser Center, M.V. Lomonosov Moscow State University, Moscow 119991, Russia; anlug@bmp.ilc.edu.ru (A.L.); avpriezz@gmail.com (A.P.); 3 Physics Department, M.V. Lomonosov Moscow State University, Moscow 119991, Russia

**Keywords:** nanodiamond, red blood cell, albumin, immune responses

## Abstract

Nanodiamonds (ND) have emerged to be a widely-discussed nanomaterial for their applications in biological studies and for medical diagnostics and treatment. The potentials have been successfully demonstrated in cellular and tissue models in vitro. For medical applications, further in vivo studies on various applications become important. One of the most challenging possibilities of ND biomedical application is controllable drug delivery and tracing. That usually assumes ND interaction with the blood system. In this work, we study ND interaction with rat blood and analyze how the ND surface modification and coating can optimize the ND interaction with the blood. It was found that adsorption of a low concentration of ND does not affect the oxygenation state of red blood cells (RBC). The obtained in vivo results are compared to the results of in vitro studies of nanodiamond interaction with rat and human blood and blood components, such as red blood cells and blood plasma. An in vivo animal model shows ND injected in blood attach to the RBC membrane and circulate with blood for more than 30 min; and ND do not stimulate an immune response by measurement of proinflammatory cytokine TNF-α with ND injected into mice via the caudal vein. The results further confirm nanodiamonds’ safety in organisms, as well as the possibility of their application without complicating the blood’s physiological conditions.

## 1. Introduction

Diamond nanoparticles, due to their nontoxicity, structural, surface and spectroscopic properties, are considered as potentially highly effective nanoparticles for biomedical applications [1]. Due to the small size (5–100 nm), nanodiamonds (ND) have a large surface area. The surface of ND crystals always contains structural defects due to the methods of their production and purification [2,3,4]; and the surface carbon atoms have unsaturated chemical bonds, which lead to the high surface activity of nanodiamonds. For bio/medical applications, the ND surface determines the ability and mechanism of interaction with objects of interest, such as cells, tissues, etc. ND also possess high sorption and chemical linking capacities, allowing target molecules and drugs for biomedical and theranostics purposes [1,5,6,7,8]. This has been recently widely discussed for ND application in biological studies and for medical diagnostics and treatment. These applications have also been successfully demonstrated in several cellular and tissue models (in vitro) [1,5,6,7,8,9]. Some recent works show that drug-nanodiamond adducts would act at the cellular level by different molecular mechanisms with respect to plant metabolite pure forms; chemical and structural modifications of nanodiamond surfaces influenced the bioactivity of the transported drugs [10,11]. However, for in vivo studies/applications of ND, the effects of toxicity and biodistribution are still some of the most important issues.

In vivo studies with non-mammalian models have demonstrated the non-toxicity nature of fluorescent ND (FND, ND obtained by the high temperature/high pressure (HTHP) method [3,4] with enhanced fluorescence [9]) and the lack of any stress response for the worm *Caenorhabditis elegans*, as well as stable observable fluorescence of the FND in the worm organs, allowing in vivo imaging and detection of the FND’s biodistribution [12,13]. While for the microorganisms *Paramecium caudatum* and *Tetrahymena thermophile* interacting with ND, especially the detonation ND (DND, [2,3]), results reveal some toxicity for higher dosages, but also these are well detectable inside and allow one to observe the biodistribution during the microorganism’s live processes [14]. The studies of ND’s influence on the early stages of living embryos were evaluated for zebrafish embryos (*Danio rerio*) [15,16]. Microinjected FND were incorporated into the dividing blastomeres [15]; FND cytoplasmic movement in the yolk cell of the embryo has been well observed; it has been shown that the FND-labeled larvae can develop into whole fishes without morphological anomalies. In the concentration-dependent study of ND injected into zebrafish embryos, in vivo toxicity evaluation on zebrafish embryos in the early developmental state shows, at a low concentration of ND, that the different stages of zebrafish embryos exhibit similar development as compared to the control groups, but increasing the concentration can affect the zebrafish embryos at the pharyngula stage [16]. Usually, FND or ND do not cause deleterious effects on the organism development. However, some toxicity of ND related to their chemical surface functionality (presence of −OH, −NH_2_ or −CO_2_H surface groups) was observed for *Xenopus laevis* embryos [17]. The study of DND (detonation nanodiamond) effects on the growth and development of chicken embryos found that ND may reduce blood vessel formation and affect the development of the circulatory system by inhibiting proangiogenic factor bFGF (basic fibroblast growth factor) [18]. This effect was used to inhibit angiogenesis in brain tumor (glioblastoma multiforme cells cultured on the chorioallantoic membrane of chicken embryo). DND application decreased tumor mass and volume, as well as vessel area [19].

Immune response studies have been performed in vivo on mammalians. No immune responses have been induced in rats, after intraperitoneal injection of ND [20]. However, injections of different kinds of DND have been observed to increase levels of blood leucocytes and changed some biochemical parameters of the mice blood [21,22]. The ND biodistribution studies have shown ND predominantly accumulated in the liver after intravenous injection in mice; spleen, kidney and lung were also target organs for ND [23]. DND after intratracheal instillation show the highest retention in the lung and are distributed mainly in the spleen, liver, bone and heart of mice [24]. Reasonably, a significant quantity of nanodiamond can be found in blood [23,24,25]. An analysis of histological morphology and biochemical parameters indicated that DND could induce dose-dependent toxicity to the lung, liver, kidney and blood [24]. From these consistent investigations, one can conclude that the biodistribution depends on the administration method [24], the size (or origin) of particles [25]; this may differ for different kinds of animals [25]. Thus, the knowledge on ND’s toxicity for living organism is still far from completed, and the roles of parameters, such as size, structure (determined by the production method), as well as surface properties, are important and deserve further studies.

Nanodiamonds have been considered ideal for in vivo imaging and applicable for cancer diagnosis. As an in vivo contrast agent, FND have been demonstrated to be useful for sentinel lymph node (SLN) mapping in mice. In the same work, the non-toxicity of FND for the mice has been confirmed [26]. To overcome the cell autofluorescence background, fluorescence lifetime imaging microscopy (FLIM) or similar time-gating fluorescence imaging techniques were proposed. It has been shown that FND, in combination with fluorescence-activated cell sorting, FLIM and immunostaining, can identify transplanted lung stem cells (LSC) and progenitor cells in histological lung sections after intravenous injection of the FND-labeled LSC into mice and track in vivo their engraftment and regenerative capabilities with single-cell resolution [27,28]. FND labeling did not eliminate the LSC self-renewal and differentiation. The combined FND-FLIM technique has been also used to monitor the temporal and spatial fates of the yolk lipoprotein-conjugated FND in vivo to trace the yolk lipoprotein transport in *C. elegans* [29].

In many animal models, ND enters the circulation and interacts with blood system. A number of studies concerning the ND interaction with blood and blood components demonstrates effects depending also on ND size, surface structure and concentrations [21,22,30,31,32]. In our previous work, we have shown that incubation of a human whole blood sample with a suspension of 100-nm HTHP ND and 5-nm DND (in vitro) does not affect the red blood cell (RBC) viability [30]. The RBC oxygenation and deoxygenation have been observed via Raman spectroscopic measurements of the hemoglobin (Hb) signal. The oxygenation/deoxygenation process was not significantly altered by ND’s interaction with the RBC, but the micro-rheological properties of RBC change after incubation with ND, in particular, the ability to deform under the action of shear stress is affected [30]. The mechanisms responsible for this effect may be based on the interaction of ND either directly with the RBC membrane or indirectly, through interaction with other blood (including plasma) components. Due to the high sorption ability of ND particles in their interaction with blood plasma, they strongly adsorb the plasma components. The adsorption of albumin, immunoglobulins, fibrinogen, insulin, etc., from blood plasma, on ND of various size and surface chemistry has been investigated [30,31,32,33,34,35]. In whole blood, the adsorption of the plasma components on ND or DND can disturb the concentrations’ equilibrium in the blood and osmolality; as a result, the hemolytic effect of DND on RBC has been pointed out [21,22,23]. Another important test is blood coagulation. It was shown that the diamond surface is less thrombogenic than a glass surface; indicating that diamond could be an attractive material for future designing of prostheses and medical devices, such as surgery instruments, blood pumps and artificial hearts [36]. The effect of ND of various sizes and concentrations on the blood coagulation parameters was studied using the activated partial thromboplastin time (APTT) test [32], which did not show the time variations relative to the coagulation initiated through the intrinsic pathway. However, in vivo studies show that ND can increase blood clotting via the activation of blood platelets and inducing thromboembolism. In the pulmonary tissue images, a high number of vessels was found occluded by platelet thrombi with ND as compared to the control [37].

Only a few works reported the interaction of ND with whole blood in vivo. Particularly, it has been shown that ultrafine DND affects some blood’s biochemical characteristics, but serious blood cell destruction was not observed [22]. Furthermore, in the same work, it has been discussed how ND can activate innate immune system cells, the formation of free radicals and correspondingly trigger the inflammation processes. Later, the effect of DND on redox and immune parameters in rats has been studied in detail upon intravenous and intraperitoneal injection of the ND [38]. It was observed that DND nanoparticles do not possess direct antioxidant characteristics, but actively contribute to the organism defense against reactive oxygen species and may also affect the inflammation process through inhibiting the activity of innate immune cells. Hematological parameters (blood cells counts) together with the state of liver cells of rats were examined in vivo after the animals received aflatoxin B1 (AfB1) alone and together with nanodiamonds synthesized by detonation [39].

In this work, we report the ND interaction with blood components and red blood cells (RBC). Along with in vitro analysis of the ND interaction in different conditions, we study small laboratory animals (rats, mice) focusing on the ND interaction with their blood, as well as analyze how the ND surface modification and coating can optimize the ND interaction with the blood. We compare the effect of ND and ND with adsorbed albumin or blood plasma components on the RBC oxygenation and deoxygenation. Serum albumins are globular proteins, the main component of blood plasma [40]. The study of the adsorption on ND of blood plasma components allows controlling spontaneous adsorption and following changes of the blood state (e.g., osmolality), controlling the interaction with blood cells (e.g., with RBC membrane) and, correspondingly, to find the condition for safe in vivo application. We also examined the immune responses of mice with ND in vivo. Finally, the in vivo and in vitro results on animal models are compared. A similar study was also conducted in vitro on human blood.

## 2. Results and Discussion

**Figure 1:**
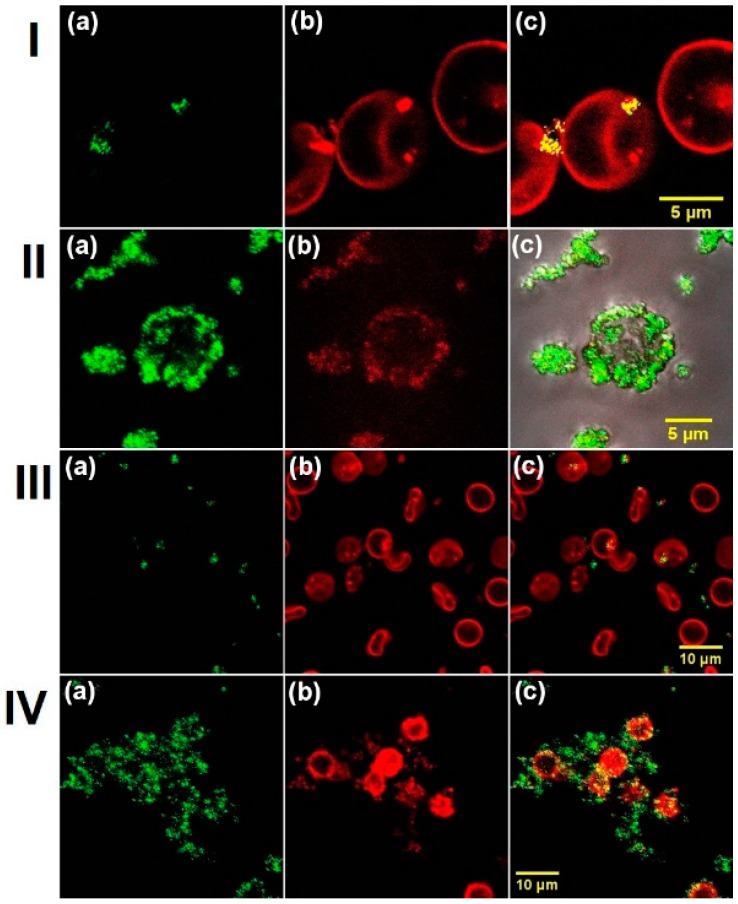
Fluorescence image of red blood cells (RBC) with nanodiamonds (ND). Human RBC with 100-nm ND (**I**: 20 µg/mL; **II**: 1000 µg/mL); rat RBC with 50 ND (**III**: 20 µg/mL; **IV**: 1000 µg/mL); (**a**) ND were excited by a 543-nm wavelength laser, and the signal was collected in the 610–640-nm region and shown in green; (**b**) the RBC’s membranes were dyed with 3,3′-dipentyloxacarbocyanine iodide (DIOC5), excited by a 488-nm wavelength laser, and the signal was collected in the 500–530-nm range, shown in red; (**c**) merged images of (**a**) and (**b**).

In this study, human and rat RBC (suspended in phosphate buffered saline (PBS) interacted with 100-nm ND and 50-nm ND at different concentrations (20 and 1000 µg/mL) for 1 h. Confocal scanning fluorescence microscopy was used to probe the location of ND. Fluorescence microscopic images of human (I, II) and rat (III, IV) RBC after the interaction are shown in Figure 1. ND fluorescence is shown in green (a); stained (after interaction with ND) RBC membrane is shown in red (b); and merged images are presented in (c). All images demonstrate adsorption of both sizes pf ND on the cell membrane. The images reveal that both ND do not penetrate inside RBC, but attach on the membrane surfaces. At a high ND concentration, the forming of ND-RBC aggregates can be observed, such as II and IV. Visually, the characteristic interaction of 1000 µg/mL ND with the RBC membrane is similar for both human and rat RBC. This concentration is much higher than most cellular models where a few µg/mL of the dosage will be applied. Previously, we have shown that ND can change the human RBC membrane’s mechanical properties and affect RBC deformability, as well as can change the rate of RBC oxygenation and deoxygenation at higher concentrations [30]. The result of the ND interaction with RBC membrane can influence the membrane structure and alter the permeability for oxygen. This can be accompanied by the changes of permeability for other small molecules, such as CO_2_, which participate in the gas exchange in the organism and do not have specific channels to go through the RBC membrane. The observed in vitro effects were weak; however, they have to be considered and minimized for in vivo use. Particularly, aggregation of nanoparticles usually causes complications in biology systems [41,42,43,44]. Both the ND aggregation itself and induced aggregation of RBC can alter the blood rheology [32]. Therefore, it is necessary to prevent the ND aggregation to minimize the side effects. The above-mentioned side effects on blood can be significantly decreased, because the ND medical applications always assume that ND has been preliminarily conjugated with the molecules of interest, such as drugs or bio-molecules for specific or non-specific interaction with the targeted system. In this study, we used a model system of ND with preliminarily adsorbed albumin. Bihari et al. [45] have established and validated a practical method to disperse nanoparticles in physiological solutions for biological studies in vitro and in vivo using albumin.

**Figure 2:**
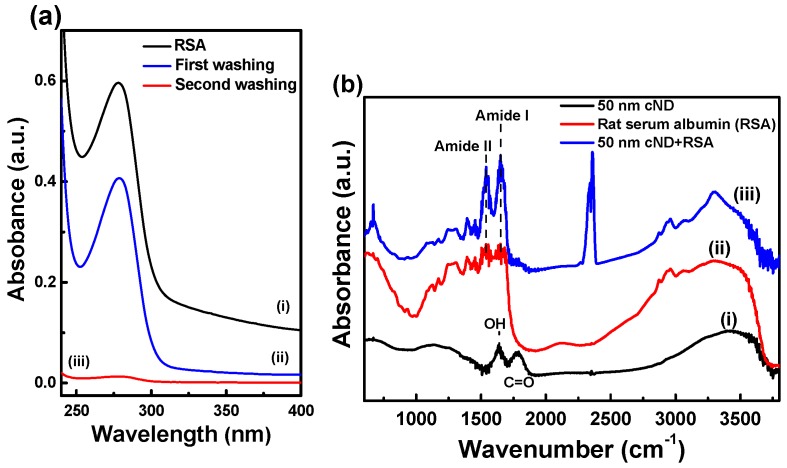
UV-visible spectra and Fourier Transform Infrared (FTIR) spectra of carboxylated nanodiamonds (cND) and ND-rat serum albumin (RSA) complex in phosphate buffered saline (PBS). (**a**) Absorption spectra of RSA solutions. In the supernatant, the RSA concentration was decreased after adsorption on ND, the first and second washing; (**b**) FTIR spectra of RSA, 50-nm cND and cND-RSA.

Albumin is the main blood plasma constituent, which is about 60% of all plasma proteins. Adsorption of blood plasma proteins, including albumin, on the ND surface has been studied previously [31]. The proteins’ adsorption on ND and related structural transformations and changes in their functional state were analyzed using UV-visible absorption and Fourier transform infrared (FTIR) spectroscopy. In the present study, rat serum albumin (RSA) of rat blood plasma was physically adsorbed on the 50-nm carboxylated nanodiamond (cND) surface, and the process of adsorption and the adsorbed RSA were analyzed. The absorption spectra of RSA solutions are presented in Figure 2a; Line (i) corresponds to the initial solution, with the original concentration of RSA; the absorption peak of the albumin is at 280 nm. After interaction with 50-nm cND, the ND-RSA complex was subjected to centrifugation and separated to measure the absorption spectra of the supernatant, Figure 2a-ii. Spectrum (iii) is the absorption of the supernatant after the second washing of the sediment in PBS and once more separated. The Spectra (i) and (ii) comparison reveals significant removal of the protein from the solution, evidencing physical adsorption on the ND surface, and the concentration of RSA in solution decreases. The value of adsorbed RSA can be estimated from the ratio between Spectra (i) and (ii). Spectrum (iii) does not show significant desorption of RSA upon washing.

The FTIR absorption spectra are depicted in Figure 2b. Spectrum (i) is from cND and shows the well-resolved C=O stretching band of the carboxyl group on the cND surface in the range of 1720–1780 cm^−1^ [46]; Line (ii) is the spectrum of RSA and exhibits the protein’s characteristic amide I, II peaks in the 1590–1700 cm^−1^ range, which are the superposition of the vibrations of peptide bonds of different conformations of the chains (turns, α-helices, β-sheets, unordered); Line (iii) is the spectrum of the ND-RSA complex displaying the amide peaks as a result of the adsorption of proteins on ND. Detailed analysis (deconvolution) of the amide I peak allows the estimation of the transformation of the proteins’ conformation [47,48], but in the present case, FTIR spectra revealed no observable transformation (analysis not shown). The result is consistent with the absorption spectra measurements and proves the RSA adsorption on the diamond surface.

**Figure 3:**
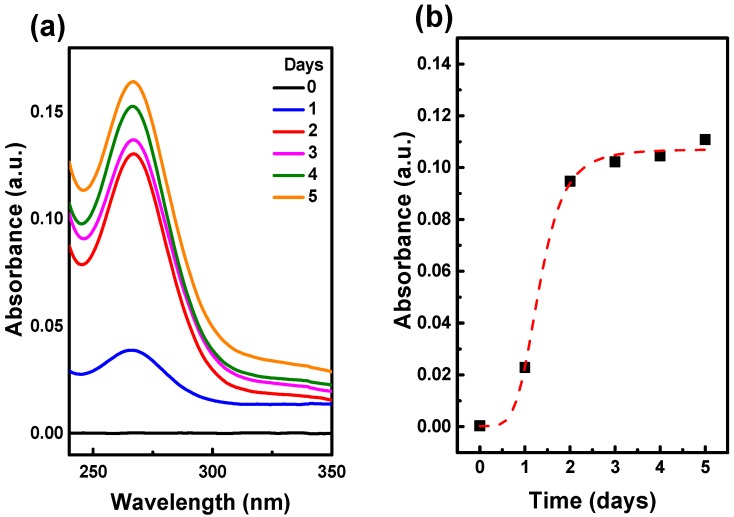
Measurement of the samples stored from 0–5 days by using absorption spectroscopy. (**a**) Time (date) dependence absorption spectra of supernatant from five-day samples; (**b**) fitting of the spectra from (**a**) at the peak around 280 nm, found RSA released and reaches saturation in two days of storage.

The time stability of the obtained 50-nm ND-RSA complex has been estimated for five days of storage. The UV-visible absorption spectra of protein released into PBS in dependence of time are shown in Figure 3a. The time dependence of the released protein as a function of day is illustrated in Figure 3b. One day after preparation, only a very small amount of protein released into PBS is observed. On the second day, protein releases more rapidly and reaches saturation on the second day. Thus, the complex stability is satisfactory for the present measurements. The complex stability and the protein release should be considered with respect to the method of physical adsorption to attach molecules of interest to ND for bio-application.

**Figure 4:**
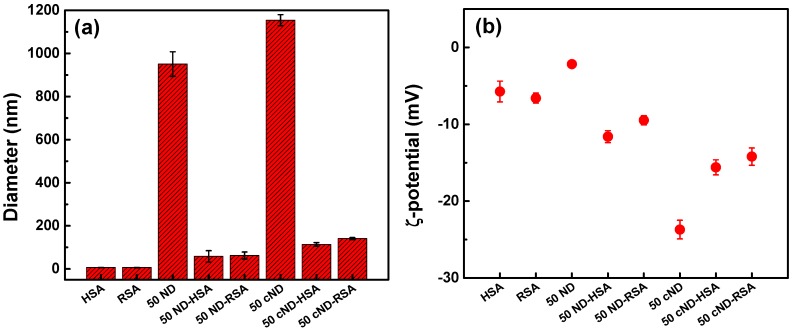
Dynamic light scattering and ζ-potential measurements of ND and ND-HSA (RSA) complexes in PBS complex in PBS. (**a**) Dynamic light scattering measurements of HAS, RSA, 50-nm ND and ND-HAS (RSA). Fifty-nanometer cND dispersed in PBS have a large aggregation size up to ~1150 nm; the average size of cND-HSA (RSA) is about 150 nm. After HAS (RSA) adsorption on the ND surface, ND is better dispersed; (**b**) The ζ-potential of HAS (RSA) is −7 mV, of 50 cND is −25 mV and of cND-human serum albumin (HSA) (RSA) is −15 mV at pH in ~7.4.

To further analyze the prepared ND-RSA, dynamic light scattering (DLS) was used to study the interaction of albumin molecules with ND in the aqueous solutions. The size and surface charge of the particles of the 50-nm cND-RSA complex were measured. Figure 4a presents the results of DLS measurements of the sizes of particles in PBS and the effect of the albumins’ adsorption. The measured hydrodynamic size of the molecules human serum albumin (HAS) or rat serum albumin (RSA) is about 7 nm; the average size of ND and cND particles before adsorption of the albumins is ~900 and ~1150 nm, correspondingly. After interaction with albumins, the particle sizes of ND or cND in complex with albumin decrease to 150–170 nm. Therefore, a high degree of aggregation of ND (or cND) in PBS is observed, but it can be significantly decreased by the adsorption of albumins on the nanodiamond surface. This indicates that albumin adsorption allows preventing ND aggregation and avoiding the side effect of blocking capillaries when ND enter circulation in blood and uncontrollable adsorption of blood components.

For the ND shown in Figure 4a, ζ-potential measurements are presented in Figure 4b. At pH 7.4, the ζ-potentials are −7 (both albumins) and −2 mV (ND) and change to −23 mV after carboxylation. This is presumably due to the carboxylic group appearing on the ND surface. Albumins’ adsorption shifts the ζ-potential of ND to −10 mV. This is another indication of protein effects due to the more electric positive nature of proteins. We can conclude that the adsorption of albumin can prevent ND aggregation, which may cause side effects on the blood rheology and plasma properties (particularly, on macromolecules composition, osmotic pressure). The effect of the ND-protein complex on other blood properties or functions is also decreased in comparison with ND. We demonstrate it for RBC, focusing on the RBC ability to transfer oxygen [30].

**Figure 5:**
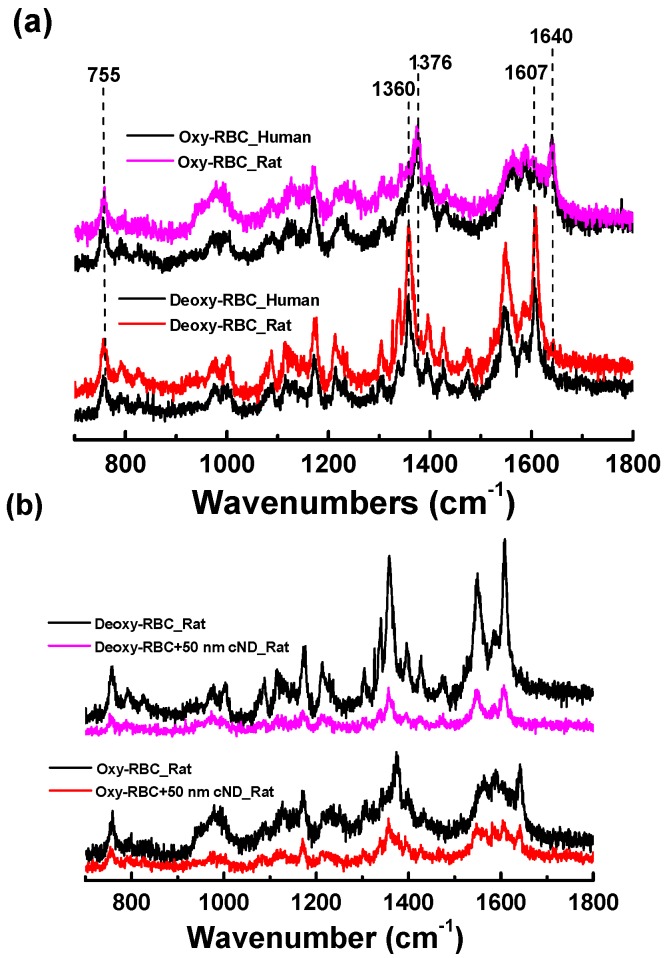
Raman spectra of human and rat RBC oxygenated and deoxygenated. (**a**) Comparison of human and rat RBC oxygenated and deoxygenated dynamics; (**b**) rat RBC without and with 50-nm cND’s oxygenated and deoxygenated dynamics; the ND concentration is 20 µg/mL.

In Figure 5a, we compare the Raman spectra of human and rat RBC in fully-oxygenated and deoxygenated states. The RBC Raman spectrum is determined by hemoglobin (Hb), the iron-containing oxygen-transport protein. Hb has a strong and complex Raman spectrum, very sensitive to the spin state of the iron atom in the functional center of Hb, heme (the spin state depends on the oxygenation state of Hb) and to conformational changes of the Hb molecules. From the Raman result, the spectrum significantly changes when Hb changes from the relaxed (R) state of oxygenated Hb to the tense (T) state of deoxygenated Hb [33,34], and Raman spectroscopy can be used for the study of Hb and RBC states and functions. As seen from Figure 5, no significant difference between Raman spectra for human and rat RBC is observed when interacting with ND. For this reason, rat models can be useful in studying the effects of ND. However, we see the influence of ND on the Hb/RBC from the Raman spectra of rat RBC with and without 50-nm cND in oxygenated and deoxygenated states, which are compared in Figure 5b. To analyze this influence, the dynamics of RBC deoxygenation and following oxygenation in the presence of ND has been observed. The oxygenation degree of the RBC sample was varied by N_2_ purging or by spontaneous interaction with air. To estimate the changes in the oxygenation degree of RBC, we used the characteristic Raman peaks for deoxygenated Hb in the T-state at 1606–1610 (ν_10_) and 1358–1360 cm^−1^ (ν_4_) and for oxygenated Hb in the R-state at 1638–1640 and 1370–1375 cm^−1^, correspondingly, assuming that the oxygenation degree is proportional to the relation *I*_oxy_/(*I*_deoxy_ + *I*_oxy_) [33]; below, we also call this the “peaks ratio” (PR).

**Figure 6:**
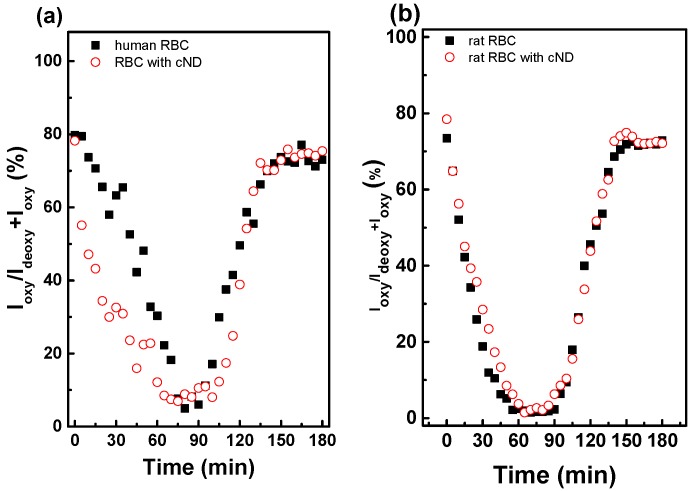
Oxygenation dynamics of rat RBC interaction with 50-nm cND. The formula $\text{PR}\left(\%\right)=\frac{I_{\text{oxy}}}{I_{\text{oxy}}+I_{\text{deoxy}}}\times 100$ is used to estimate oxygen saturation changes (PR, peaks ratio). Oxygenation dynamic curves are shown in (■) for RBCs and (○) for RBCs with 50-nm cND. (**a**) Human RBC interaction with 50-nm cND; (**b**) rat RBC interaction with 50-nm ND. The ND concentration is 20 µg/mL.

The results obtained using ν_10_ and ν_4_ modes for the estimation of oxygenation degree were similar. The changes of this value in the process of deoxygenation by purging N_2_ and following spontaneous oxygenation in the air for human and rat RBC are compared. The oxygenation and deoxygenation dynamics of RBC with 50-nm cND is shown in Figure 6. The dependences of *I*_oxy_/(*I*_deoxy_ + *I*_oxy_) on time for human and rat RBC at a relatively low concentration of ND (20 µg/mL) are comparable: deoxygenation takes about 70 min for human RBC, about 60 min for rat RBC; the oxygenation of both is 40–45 min. The relative changes of the value are about 80% in both cases.

**Figure 7:**
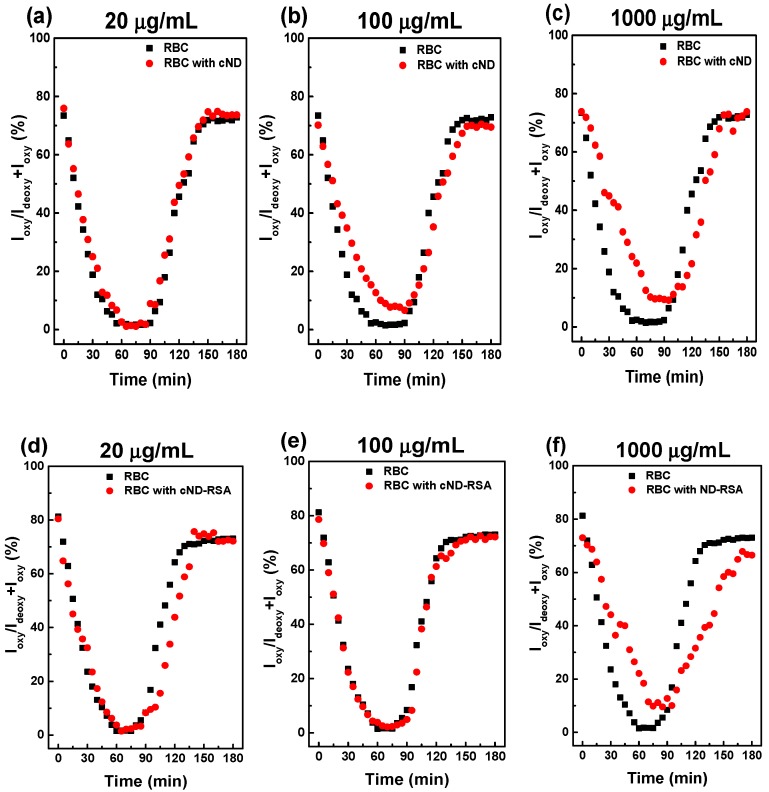
Oxygenation dynamics of the rat RBC interaction with 50-nm cND (**a**–**c**) and cND-RSA (**d**–**f**) in various concentrations. (**a**,**d**) 20 µg/mL; (**b**,**e**) 100 µg/mL; (**c**,**f**) 1000 µg/mL. The formula $\text{PR}\left(\%\right)=\frac{I_{\text{oxy}}}{I_{\text{oxy}}+I_{\text{deoxy}}}\times 100$ is used to estimate oxygen saturation changes. Oxygenation dynamic curves are shown in (■) for RBCs and (●) for RBCs with 50-nm cND or cND-RSA.

Rat RBC are slightly sensitive to ND’s presence, as one can see from Figure 5 and Figure 6, as well as from a comparison of Figure 7 and with previous results for human RBC [30]. The effects of 50-nm cND on the rate of RBC deoxygenation-oxygenation are concentration dependent. In Figure 7, the deoxygenation-oxygenation dynamics are compared for rat RBC treated with 50-nm cND in different concentrations (a–c). The same concentrations are compared in Figure 7d–f for the interaction in the presence of the 50-nm cND-RSA complex with rat RBC. For an ND concentration of 20 µg/mL (Figure 7a), no significant effect of ND is observed. The effect becomes more significant for an ND concentration of 100–1000 µg/mL for rat RBC and for an ND concentration of 1000 µg/mL for human RBC; the delay of oxygenation is observed in these cases. Notice that for the ND with adsorbed RSA, the aggregation is decreased (Figure 4), and the effect on the oxygenation/deoxygenation is not affected up to 100 µg/mL (Figure 7e).

**Figure 8:**
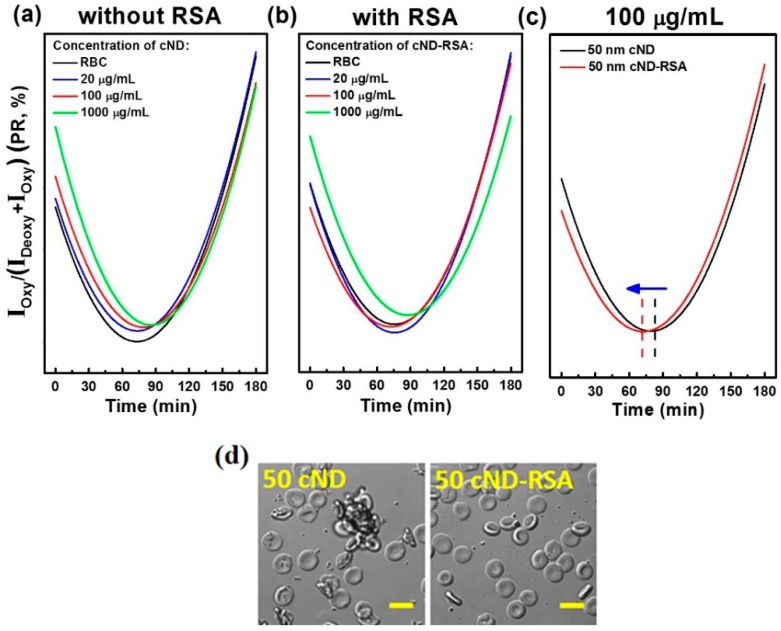
Oxygenation dynamics of the rat RBC interaction with 50-nm cND and cND-RSA in various concentrations. The peak intensities of the Raman bands for oxygenated (*I*_oxy_) and deoxygenated (*I*_deoxy_) are compared. The peak ratio (PR) was calculated using $\text{PR}\left(\%\right)=\frac{I_{\text{oxy}}}{I_{\text{oxy}}+I_{\text{deoxy}}}\times 100$, and the dependencies of PR on time during deoxygenation and following re-oxygenation were fitted using the standard second order polynomial fit. The results of fitting are shown: (**a**) the rat RBC interaction with various concentrations of 50-nm cND; (**b**) the rat RBC interaction with various concentrations of 50-nm cND-RSA; (**c**) comparison of the rat RBC interaction with 100 µg/mL 50-nm cND and 50-nm cND-RSA; the optical images are shown in (**d**); the scale bar is 6 µm.

The results in Figure 7, the dependencies of the peaks ratio (in %) on time during deoxygenation and following oxygenation, were fitted using the standard second order polynomial fit, and the fitting is plotted in Figure 8. Figure 8 shows the dynamics of the rat RBC deoxygenation-oxygenation interaction with 50-nm cND (a) or the cND-RSA complex (b) for various concentrations. Comparing Figure 7a–c and Figure 7d–f, one can observe that coating ND with blood plasma proteins significantly decreases the observed ND effect on the degree of deoxygenation for the middle ND concentration (Figure 7b,e, 100 µg/mL). As for the characteristic times of oxygenation and deoxygenation, for the results of fitting for RBC with 100 µg/mL ND, shown in Figure 8c, a delay of reoxygenation of about 15 min is observed for the interaction with cND. At a high concentration (1000 µg/mL), adsorption of RSA on the ND does not decrease the ND effect on the deoxygenation-oxygenation dynamics. Presumably, this is due to a high concentration blocking the oxygen entrance to the RBC. The optical images of rat RBC interacting with 100 µg/mL of cND and cND-RSA are shown in Figure 8d. The result confirms that after RSA adsorption on the ND surface, ND re better dispersed in the PBS (in agreement with Figure 3a) and do not case the well-observed RBC aggregation.

**Figure 9:**
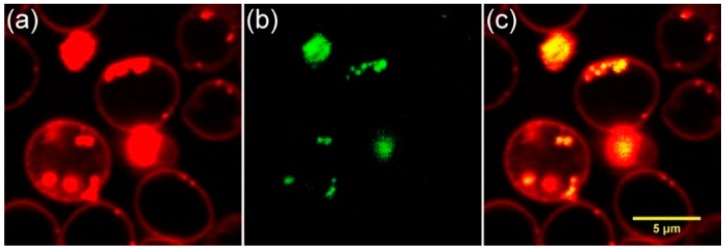
Confocal images: in vivo circulation of nanodiamonds in rat. (**a**) The stained RBC membrane is shown in red; (**b**) ND fluorescence is shown in green; and (**c**) the merging of (**a**) and (**b**).

ND interactions with blood circulation in the animal model were tested in vivo. For this measurement, 100-nm ND were injected in caudal vein (c.v.) of rat. After 30 min of ND circulation in the blood system, blood was withdrawn and dissolved in PBS (1:200) for confocal laser-scanning microscopic imaging. The obtained images are shown in Figure 9. The RBC’s membrane, dyed with 3,3′-dipentyloxacarbocyanine iodide (DIOC5), is shown in red (Figure 9a); the ND fluorescence excited by a 543-nm wavelength laser and detected in the 610–640-nm range is shown in green (Figure 9b). Figure 9c is the merged image of (a) and (b). The result reveals that ND have attached to RBCs and remain in blood circulation for 30 min. This means that ND can remain in blood circulation for several cycles of the blood circulation in the rat body without been cleared. This shows great promising applications, such as using ND as a vehicle for drug delivery.

**Figure 10:**
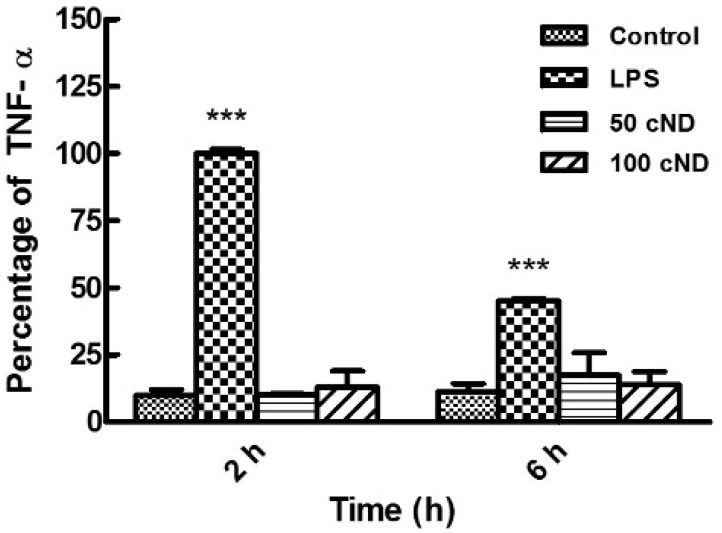
In vivo test of TNF-α activation in the blood system of mice after injection of cND. The cND were suspended in PBS with concentration 1 mg/mL and injected into mice via the caudal vein (c.v.) route in a quantity of 20 mg/kg of body weight; LPS at a concentration of 1000 ng/kg of body weight (b.w.) was used as a positive control. Blood samples were collected via the orbital sinus of the mice at 2 and 6 h after injection. *n* = 9 (three experiments repeated three times); *** *p* < 0.001; significant amelioration versus respective control groups. Data are the mean ± SD.

When the immune system senses a substance being introduce, the synthesis of proinflammatory cytokines and chemokines is activated. The blood cells, such as lymphocytes and neutrophils, as well as the surrounding tissues’ cells (e.g., macrophages) are involved in the immune response. The level of proinflammatory cytokines and chemokines characterizes the immune response of the organism (or the 4 model system) to the intrusion [49]. To understand if ND injected and circulating in blood induce the immune response in vivo, the level of proinflammatory cytokine TNF-α, one of the agents in the chain of the immune response, involved in systematic inflammation [50], was analyzed. The ND suspension was injected into mice via caudal vein (c.v.). While ND circulate in the blood, blood samples were collected after 2 and 6 h, and the in vivo production of TNF-α was tested. Figure 10 demonstrates the TNF-α level in the blood plasma. One can see that the TNF-α production was not activated by ND in comparison to the control (treatment with PBS). As a positive control, lipopolysaccharide (LPS), from the outer membrane of Gram-negative bacteria, was injected to activate the immune response. LPS is the key inflammatory component of Gram-negative bacteria, which promotes the secretion of pro-inflammatory cytokines. In Figure 10, an increased TNF-α level after treatment with LPS is observed in contrast to ND treatment. Thus, an ND-induced immune response has not been observed for the ND in vivo application.

## 3. Experimental Section

### 3.1. Nanodiamond Carboxylation

Synthetic diamond powders with an average size of 50 and 100 nm (Kay Diamond, Boca Raton, FL, USA) were carboxylated/oxidized to remove the surface non-diamond fractions and impurities, as described before [46]. In short, as received ND were treated with a mixture of concentrated H_2_SO_4_ and HNO_3_ (ration 3:1) at room temperature for 24 h, following separation with centrifugation and washing with bi-distilled water to remove surface non-diamond fractions and impurities. The carboxylation, the forming of COOH surface groups, was confirmed via Fourier transform infrared spectroscopic measurements (ABB Bomem MB154 FTIR spectrometer, Switzerland). The characteristic C=O (1720–1780 cm^−1^) and O–H (1620–1640 cm^−1^) bands of the carboxyl groups were observed. ND particle size and ξ-potential were estimated using the dynamic light scattering method (DLS) with a Zetasizer Nano ZS (Malvern Instruments, Malvern, UK).

### 3.2. Blood Sample Preparation

Fresh human blood was obtained from a healthy volunteer and was transferred into an ethylenediaminetetraacetic acid (EDTA)-covered tube. The research methods were approved by the Research Ethics and Use Committee (REC) of Buddhist Tzu Chi General Hospital (Approval REC No. IRB101-149). For the rat red blood cell (RBC) suspension preparation, 3 mL of whole blood were withdrawn from the tail of Wistar rats and transferred into EDTA-covered tubes. The research methods were approved by the Animal Care and Use Committee of National Dong Hwa University (Approval ID100004). The rats were narcotized for the experiments. The RBC were separated from the human blood using centrifugation with a speed of 1500 rpm (250× *g*) for 5 min at 4 °C. RBC mass was further separated from the supernatant plasma and then was washed with standard phosphate buffer saline (PBS: 4 g NaCl, 0.1 g KCl, 0.72 g Na_2_HPO_4_ and 0.12 g KH_2_PO_4_ were dissolved in 450 mL of bidistilled H_2_O; the PBS pH value was 7.4 ± 0.02). Concentrations of PBS to RBC were 2:1 at 4 °C, and the pH was maintained at the value of 7.4. The RBC + PBS mixture was centrifuged at a rate of 3500 rpm for 1 min. This process was repeated 3–5 times. Finally, the washed RBC were diluted with PBS at the ratio of 5:1000 µL (RBC:PBS).

### 3.3. Albumin Adsorption on Nanodiamond (ND)

Human serum albumin (HSA, Sigma, Ronkonkoma, NY, USA) and rat serum albumin (RSA, Sigma) were physically adsorbed on ND. The method has been described in detail previously [31,45]. In short, the albumin solution with a concentration of 40 mg/mL was mixed with an aqueous suspension of ND with a concentration of 4 mg/mL in the ratio 1:1. After thorough mixing for two hours, the mixture was centrifuged, and ND with the protein adsorbed on the surface were separated. The sediment was washed twice in distilled water to remove the non-adsorbed protein in the solution or protein weakly bound to the ND surface. This method ensures the formation of a stable ND-protein complex. To estimate the adsorption, the absorption spectra of protein solutions before adsorption and after interaction with ND and separation of ND-protein complex were measured using a UV-visible spectrometer (Jasco V-550, Tokyo, Japan). The ND-protein complex is then further confirmed via measurements of the size and ζ-potential of ND and the ND-protein complex (at pH = ~7.4), using a Zetasizer Nano ZS (Malvern Instruments, Malvern, UK).

A similar method is used to prepare the ND complex with whole plasma components; the details has been described before [30]. In brief, whole blood was withdrawn from human or Wistar rats with the use of EDTA as the anticoagulant. The plasma fraction was separated by centrifugation at 1500 rpm for 5 min at 4 °C. For the interaction of ND with plasma, 2 mg of cND were dispersed in 1 mL of plasma and vortexed at 37 °C for 2 h, then centrifuged and washed to separate the unreacted plasma components present in the supernatant. The sediment was dispersed in 1 mL of standard phosphate buffer saline. For the cND-RSA complex stability test stored in PBS solution, five samples of cND-RSA were prepared at the same time and stored in a refrigerator at 4 °C. Every day, for 5 days, one sample was taken for measurements. The sample was centrifuged at 11,000 relative centrifugal force (rcf) for 10 min, then the absorption spectrum of supernatant was measured to estimate the protein desorption.

### 3.4. Microscopic Investigation of the Interaction of Nanodiamonds and the Nanodiamond-Albumin Complex with Red Blood Cell (RBC)

The 50- and 100-nm ND and ND-HSA and ND-RSA were used to observe and visualize the interaction of ND/ND-albumin with RBC. For imaging of RBC with nanodiamonds in vitro, the ND were suspended in PBS at a 10-mg/mL concentration. From this suspension, 5 and 280 µL of the ND + PBS suspension were mixed with 1500 µL of PBS and added to the 1005-µL RBC + PBS suspension. The final concentration of cND in the prepared cND + RBC + PBS sample used for the measurements was ~20 and 1000 µg/mL at pH 7.4.

For imaging of rat RBC with nanodiamond in vivo, 3–4 month-old male white rats weighing 380–520 g were used. Rats were narcotized during the experiments. The 100-nm ND concentration is 5 mg/kg of body weight (b.w.). The ND water suspension with 0.9% NaCl was prepared with the use of an ultrasound sonicator and heated up to 37 °C before injecting; the volume of the injection was 0.3 mL per 500 g of body weight. ND were injected into the rat’s caudal vein. The control group of rats received a similar volume of 0.9% NaCl solution. Rat blood was drawn for the fluorescence imaging measurements 30 min after injection. The solution of EDTA (2 mg/mL) was used as a stabilizer. The blood was put into an EDTA-covered tube and diluted with 0.9% NaCl solution in a concentration of NaCl:blood of 1:3 at pH 7.4.

Fluorescence and optical (bright field, BF) images of the ND interaction with the RBC were observed using a scanning confocal fluorescence microscope (TCS-SP5, Leica, Wetzlar, Germany). To observe the fluorescence signal from the 5- and 100-nm-sized ND, the excitation wavelengths used were 488 and 543 nm; the corresponding signal detection was between 491–539 nm and 562–659 nm. The wavelengths were selected according to the fluorescence spectra observed for the ND [51,52]. Fluorescence from the nitrogen vacancy (NV^0^) defect centers, centered near 605 nm (in the present case, excited with a 488-nm wavelength laser) and NV^−^ centers’ fluorescence band centered at 670 nm (excited with 543 nm) were used. It is to be mentioned that Hb fluorescence at these excitations was also observed. Confocal scanning was also performed along the *Z*-axis to probe the localization of ND relative to the RBC. To observe the fluorescence signal from 50-nm ND, the excitation wavelength was 633 nm, and the corresponding signal was detected in the range 643–700 nm. No emission from Hb is observed at that excitation. Confocal scanning was also performed along the *Z*-axis to probe the localization of ND relative to the RBC. For that, the RBC membrane is dyed with DIOC5 (Invitrogen, Waltham, MA, USA); the fluorescence of DIOC5 was excited at 488 nm, and the emission was collected in the range 500–515 nm.

### 3.5. Raman Spectroscopic Measurements

A T64000 (Jobin Yvon, Longjumeau, France) Raman spectrometer with a 532-nm excitation wavelength laser was used to characterize the ND. For 100-nm ND, a pronounced characteristic peaks of sp^3^ bonded carbon (diamond structure) at 1332 cm^−1^ dominates. The spectra of 50 ND show a decreased widened peak with a shift to 1322–1325 cm^−1^, which is usually observed due to the phonon confinement effect at decreasing crystalline size to a few nm. Raman peaks were observed on the background of ND fluorescence spectra, measured with 532-nm (T64000) and 488-nm (spectrometer a-SNOM, Witec, Ulm, Germany) wavelength excitation lasers. These excitations excite diamond NV^0^ and NV^−^ defect centers, with fluorescence centered near 605 nm (with a 488-nm wavelength laser) and at 670 nm (with a 532-nm wavelength laser) correspondingly [51].

The 50- and 100-nm ND and ND-HSA and ND-RSA were used to study the interaction of ND/ND-albumin with the RBC of human or rat blood and ND’s effect on the oxygenation-deoxygenation process of RBC, respectively. ND were suspended in PBS at a concentration of 10 mg/mL. Five, 25 and 280 µL of the ND + PBS suspension were mixed with 1500 µL of PBS and added to the 1005-µL RBC + PBS suspension. The final concentration of ND in the prepared ND + RBC + PBS sample used for the measurements was ~20, 100 and 1000 µg/mL.

The interaction of ND and ND-HSA (or ND-RSA) with RBC and ND’s effect on the oxygenation-deoxygenation process of RBC were studied using Raman spectroscopic analysis of the Hb spectra, as described previously [28]. The Raman spectra of RBC were measured using a Raman spectrometer (T64000, Jobin Yvon) with a 532-nm excitation wavelength laser. RBC spectra are determined by the Raman signal of Hb during one cycle of deoxygenation-oxygenation for a single RBC, and each experiment was repeat for 5–10 RBC.

The oxygenation degree of the samples was monitored using the oxidation state marker Raman bands. In this work, the intensities of the bands at 1640 cm^−1^ (oxygenated state) and 1607 cm^−1^ (deoxygenated state), or at 1376 and 1360 cm^−1^, correspondingly, were the ratio to the band at 755 cm^−1^, which is neutral to the oxygenation degree and can be used as a reference point. These band intensities (*I*) give the estimation of the changes of the oxygenation degree, which is proportional to *I*_oxy_/(*I*_deoxy_ + *I*_oxy_) [33,53]. The Raman spectra were measured every 5 min. The RBC suspension in the PBS was placed in a culture dish for the purging of nitrogen. Oxygenation was achieved in ambient atmospheric pressure, and deoxygenation was performed with nitrogen gas purging (10 kg/cm^2^).

### 3.6. Measurements of Tumor Necrosis Factor α (TNF-α) in Vivo Production

Eight week old male C57BL/6 mice (*n* = 9; three experiments repeated three times) weighing 22–24 g were used. The mice were purchased from the National Laboratory Animal Center and housed in the Laboratory Animal Center, Tzu-Chi University (Hualien, Taiwan). The mice underwent procedures of anesthesia with an intraperitoneal injection of ketamine:xylazine (80:10 mg/kg body weight). The research methods were approved by the Animal Care and Use Committee of National Dong Hwa University (Approval ID100004). The mice were narcotized for the experiments. The 50- and 100-nm cND were suspended in a concentration of 1 mg/mL PBS, sonicated for 3 min and injected into mice via the caudal vein (c.v.) route with 20 mg/kg of body weight (b.w., the volume of the injection was 20 µL per 1 g of b.w.) or LPS in a concentration of 1000 ng/kg of b.w. The animals of the control group received a similar volume of PBS solution. Blood samples were collected via orbital sinus of the mice at 2 and 6 h after injection. The mice blood samples were immediately placed on ice, and within 10 min, the plasma was collected using centrifugation at 400× *g* for 10 min and aliquoted for storage at −80 °C until measurements. Mice blood serum was assayed for TNF-α levels by the AlphaLISA Mouse Tumor Necrosis Factor alpha kit (mTNF-α, Blossom biotechnologies Inc., Taipei, Taiwan). Initially, AlphaLISA Immunoassay buffer was used to prepare standard dilutions with concentrations of 100,000, 30,000, 10,000, 3000, 1000, 300, 100, 30, 10, 3, 1 and 0.3 pg/mL; then, 5 µL of standard dilutions and mice serum were added to the 384-well AlphaPlate microplate. Subsequently, 20 µL of 25 µg/mL AlphaLISA anti-mTNF-α acceptor beads and 2.5 nM of biotinylated antibody anti-mTNF-α mixtures were added to each well and then incubated at room temperature for about 60 min. After incubation, 25 µL of streptavidin donor beads (concentration is 80 µg/mL) were added to each well and incubated at room temperature for 30 min in the dark. After 30 min of incubation, the optical density (O.D.) with the absorbance at 615 nm was measured by the EnSpire^®^ Multimode Plate Reader (PerkinElmer, Santa Clara, CA, USA) [50].

## 4. Conclusions

In this study, the interaction of 50- and 100-nm ND with human and rat RBC is presented. The surface and optical properties of the ND adsorbed with blood proteins were analyzed using UV-Vis and FTIR spectroscopy. The effective size and surface charges of both ND and ND with serum albumin are obtained. UV-Vis spectroscopic studies reveal that the serum albumins’ desorption from the ND surface is stable after two days. Raman spectroscopy was used to probe the oxygenation states of a single red blood cell. The RBC can be repeatedly oxygenation/deoxygenated in vivo while the Raman spectra were measured. The results show that with the adsorption of ND, it does not affect RBC’s oxygenation state at lower concentrations, below 100 µg/mL. The adsorption of serum albumins largely reduced the aggregation of ND, also retaining the oxygenation properties for RBC below 100 µg/mL, both for human and rat RBC.

In the in vivo animal model, injected in blood, ND attaches to the RBC membrane and circulates with blood for at least 30 min. The immune response of the organism to 50- and 100-nm cND was tested in vivo by the measurement of proinflammatory cytokine TNF-α production. This parameter does not indicate the immune response stimulation by ND. These results suggest that nanodiamonds are safe for a number of processes in organisms and confirm the possibility of their application without complicating the blood’s physiological conditions.
